# Breastfeeding practices and determinants of exclusive breastfeeding in a cross-sectional study at a child welfare clinic in Tema Manhean, Ghana

**DOI:** 10.1186/s13006-018-0156-y

**Published:** 2018-03-06

**Authors:** Bernard Yeboah-Asiamah Asare, Joyce Veronica Preko, Diana Baafi, Bismark Dwumfour-Asare

**Affiliations:** 1 Researchweb Africa, P. O. Box 2591, Sunyani, Brong-Ahafo Region Ghana; 2 Tema General Hospital, P. O. Box 14, Tema, Greater Accra Region Ghana; 3 Sunyani Municipal Hospital, Private Mail Bag, Sunyani, Brong-Ahafo Region Ghana; 4 Department of Environmental Health and Sanitation Education, College of Agriculture Education, University of Education, Winneba, Asante-Mampong, Ghana

**Keywords:** Exclusive breastfeeding, Infant feeding, Breastfeeding, Sociodemographic, Ghana

## Abstract

**Background:**

Exclusive breastfeeding is important for child health and growth, but its practice is low in many developing countries. This study aimed at determining the breastfeeding practices and examining the sociodemographic characteristics that influence exclusive breastfeeding among mothers attending child welfare clinic at Manhean, in the Tema East Sub-Meteropolitan area of Greater Accra region of Ghana.

**Methods:**

This was a cross-sectional study that employed a structured questionnaire to collect data among 355 mothers of children aged 0–24 months selected through simple random sampling, attending a child welfare clinic from May to June, 2016. Breastfeeding practices were assessed based on the practices in the last 24 h prior to the study as defined by the World Health Organization.

**Results:**

There was a universal awareness and high knowledge about exclusive breastfeeding among mothers, but prevalence among infants less than 6 months was 66.0% (*n* = 138/209). Mothers currently breastfeeding were 263 (74.0%); 225 (63.4%) initiated breastfeeding within the first hour after delivery and 289 (81.0%) of the mothers offered colostrum to babies after delivery. Continued breastfeeding rate at 1 year was 77.3% (*n* = 17/22). Only 33.7% (*n* = 31/92) of infants aged 6–8 months had started receiving complementary foods. For infants aged less than 24 months, 30.1% (*n* = 98/326) were bottle feeding. Mothers aged 20–24 (Adjusted odd ratio [AOR] 9.80; 95% confidence interval [CI] 2.11, 45.46), 25–29 (AOR 9.49; 95% CI 2.07, 43.47) and 30–34 (AOR 6.02; 95% CI 1.41, 25.65) were more likely to practice exclusive breastfeeding. Mothers who had tertiary education were less likely to practice EBF than those with no education (AOR 0.18; 95% CI 0.36, 0.85). Mothers from ethnic groups in northern Ghana were less likely to exclusively breastfeed their infants compared to those of Ghanaian (Ga) ethnicity (AOR 0.29; 95% CI 0.09, 0.96).

**Conclusions:**

Exclusive breastfeeding and timely complementary feeding practices are suboptimal. Educational status, age and ethnicity of mothers strongly predicted maternal practice of exclusive breastfeeding. Interventions emphasizing a practical education should therefore be targeted at addressing factors that influence exclusive breastfeeding.

## Background

Optimal breastfeeding practice decreases child death and contribute significantly to the long term health of children [1]. In 2016, a Lancet series estimated that 823,000 deaths of children under five years could be prevented every year through optimal breastfeeding practices [2]. Optimal breastfeeding practices reduce hospitalization among children from diarrhoea, respiratory infections, and otitis media illnesses [2].

In view of the benefits of breastfeeding, starting breastfeeding in the first hour of delivery, exclusive breastfeeding (EBF) for the first 6 months of life and continued breastfeeding together with suitable complementary foods for up to 2 years or beyond are recommended as the best infant feeding plan for optimal growth, development and health [1]. Yet just about half of the 80% of neonates who are given breast milk worldwide initiated breastfeeding in the first hour of birth, and late initiation of breastfeeding is still a challenge in developing countries [2]. The rates of continued breastfeeding also appear to be declining among poor mothers [2]. Exclusive breastfeeding is reportedly low in many countries [3–7]. Globally, just 38% of infants less than 6 months are exclusively breastfed [8]. The trend of exclusive breastfeeding among infants less than 6 months in developing countries has taken over a decade to increase from 33% in 1995 to 39% in 2010 [9].

In Ghana, breastfeeding is common with nearly all children being breastfed. However, the Ghana Demographic Health Survey in 2014 has reported an exclusive breastfeeding rate of 52% at 6 months [10], which is below the optimal EBF rate of 90% in infants less than 6 months set by the WHO/UNICEF for developing countries [11].

Among factors identified to influence breastfeeding practices in both developing and developed countries are sociodemographic factors [4, 12–15]. Findings from a number of studies have emphasized maternal education [5, 16] and in the study by Vieria and colleagues, mothers who had an education for 8 years or less were at 34% higher risk of stopping exclusive breastfeeding [16]. A study in Nigeria has found that mothers who visited the antenatal clinics were positively associated with EBF and mothers who resided in rural areas were less likely to practice exclusive breastfeeding [17]. Seid et al. have reported in Ethiopia that being a housewife, having prenatal EBF plan, giving birth vaginally and receiving infant feeding counseling was associated with the practice of exclusive breastfeeding [18]. Findings from another study in Ethiopia has observed that mothers with less income status are positively associated with practice of exclusive breastfeeding [19]. Mothers who are unemployed are also positively associated with the practice of exclusive breastfeeding [20]. Furthermore, mothers living in their own homes and the place of delivery have also been documented to predict the practice of exclusive breastfeeding [21]. In Ghana, a study has highlighted that mothers who delivered at the government hospitals were at a higher odd of practicing exclusive breastfeeding [22]. Child factors such as the age and sex of child have also been indicated to predict the practice of exclusive breastfeeding [20, 23]. In Nigeria, Agho and colleagues have reported that female infants are more likely to be exclusively breastfed than their male counterparts and also infants aged less than 2 months are more likely to be exclusively breastfed [17].

There are enormous benefits of breastfeeding to both mothers and infants, yet the levels of breastfeeding practices particularly in developing countries including Ghana continue to be suboptimal. Therefore, understanding the breastfeeding practices and the factors that predict them is important in designing and carrying out successful interventions. This study aimed at determining the breastfeeding practices and to examine the sociodemographic factors associated with exclusive breastfeeding among mothers attending child welfare clinic at Manhean Health Center in the Tema Metropolitan area of the Greater Accra region in Ghana.

## Methods

### Study design and setting

This was a cross-sectional descriptive study carried out at the Child Welfare Clinic (CWC) of the Manhean Health Centre in the Tema East Sub-Metropolitan area of the Greater Accra region, Ghana. The Tema East Sub-Metropolitan is one of the three subdistricts of the Tema Metropolitan Assembly, an industrial enclave within the Greater Accra region. Manhean is one of the towns of the eleven community electoral areas of the sub-metropolitan namely; Manhean, Ashiboy, Padmore, Kwesi Plange, Bankuman, Dadeagbo, Harbour, Industrial Area, Oklor Kofi, Oninku, and Sea light.

Tema East Sub-Metropolitan area has an estimated total population of 86,136, including 18,800 women in the fertile age range of 15–49 years and 10,078 infants aged 0–24 months [24]. Most of the population belong to the Ga ethnicity and speak Ga local dialect. The Manhean town has a fishing harbour and the main occupation of the people is fishing (fish mongers and petty trading). Manhean Health Centre is located on the Eastern sector of the fishing harbour and serves as the district health centre for the Sub-Metropolitan. There are also Community Health Based Planning Services (CHPS) compounds in all communities of the eleven electoral areas. The CHPS is a community health post which focuses on the health needs of the community to improve access to equity basic healthcare. CHPS is managed by a community health officer as the leader and supported by community volunteers to enhance community participation in the delivery of primary healthcare [25]. CHPS provide services including treatment of minor ailments-malaria, acute respiratory infection, diarrhoea, and other childhood illness, as well as provide family planning services, immunizations, supervising births, antenatal and postnatal care, and health education [26].

### Sampling

The study involved mothers, attending Child Welfare Clinic at Manhean Health Centre. Mothers aged 15–49 years and were breastfeeding at the time of study and/ or had previously practised breastfeeding, with infants and young children aged 0–24 months were included in the study. The sample size was calculated using the Epi Info version 7 software based on the presumed parameters: proportion of mothers who were reported to be practising appropriate infant feeding or had knowledge of such was estimated to be 60% [27], the population of infants and young children aged 0–24 months (in the district) of 10,078, the acceptable sampling margin of error of 5% (0.05), and a confidence level of 95%. The sample size was estimated to be 355. The 355 mothers were by simple random sampling technique selected from a list of 496 mothers who were registered for child welfare services at the Manhean Health Centre. Numbers were first assigned to the names of mothers in the register, the numbers were individually written on pieces of papers and folded into small paper balls to conceal the numbers and then placed in a box. The paper balls were thoroughly mixed and all the 355 participants were randomly selected one after the other. Twenty mothers who were picked for the study and were not available for the data collection were replaced by mothers who immediately followed them in the register.

### Data collection

Four community health nurses were recruited and given practical training on the objectives of the study and data collection procedures to support as data enumerators. A structured questionnaire was then administered to the study respondents through face to face interviews from May to June 2016 by the four community health nurses during mothers’ visits to the CWC. The CWC at Manhean Health Centre is organized once a week for different sets of mothers for four times in a month. Every week 124 mothers are booked to attend their mandated one CWC visit per month. Mothers selected were interviewed as they took their turn to attend the CWC. Each interview took an average of ten minutes.

The questionnaire captured data on breastfeeding practices. Mothers were asked if they have in the past 24 h breastfed to provide information on current breastfeeding. Mothers were asked to recall how soon after birth their last babies were given breast-milk and whether they were fed with the first breast milk to gather information on early initiation of breastfeeding and colostrum. Information on current infant feeding practices (continuous breastfeeding, exclusive breastfeeding, complementary feeding, and bottle feeding) were obtained by asking the mothers of all foods or liquids given to the infant/young child in the last 24 h before the interview and whether feeding was done using feeding bottle in order to reduce recall bias. The questionnaire also collected information on sociodemographic characteristics, type and place of delivery, and maternal knowledge on exclusive breastfeeding. Maternal knowledge on EBF was assessed based on questions including ever heard of EBF, EBF entails feeding an infant with only breast milk, when to start breastfeeding after delivery, duration of EBF, the age at which a baby should be given water, and/or liquid/solid foods, and their sources of information on exclusive breastfeeding.

The questionnaire was formulated from literature reviewed on breastfeeding and was pretested at Lashibi, a community of similar settings to Manhean, in the Tema West Sub-Metropolitan area of the Greater Accra region, Ghana.

### Data analysis

Data collected were entered and analyzed using STATA version 12 (Stata-Crop, College Station, TX, USA). Apart from descriptive statistics, univariate logistic regression analysis was carried out to find the association between independent variables (sociodemographic data, type and place of delivery) and with a key infant feeding indicator; exclusive breastfeeding. Exclusive breastfeeding was selected because of its significant potential of ensuring child survival by preventing gastrointestinal infections [1].

Multiple regression analysis was done for variables that were significantly associated at the univariate logistic analysis, reporting adjusted odd ratios (AOR). Variables that were significant at *p* < 0.05 at the univariate logistic analysis were included in the multiple regression model. The significant level of 5% was set for all statistical procedures.

Breastfeeding practices were assessed based on the World Health Organization indicators for assessing infant and young child feeding practices [28]. Infant feeding practices assessed in this study were defined as follows;

- **Early initiation of breastfeeding:** Proportion of children born in the last 24 months who were put to the breast within one hour of birth
- **Exclusive Breastfeeding under 6 months**: the proportion of infants 0–5 months of age who were exclusively breastfed in the last 24 h
- **Continuous breastfeeding at 1 year**: Proportion of children 12–15 months of age who are fed breast milk in the last 24 h
- **Timely complementary feeding**: Proportion of infants 6–8 months of age who were breastfeeding and receiving solid, semi-solid or soft foods
- **Bottle feeding**: the proportion of infants less than 24 months of age who were receiving any food or drink from a bottle in the last 24 h.

## Results

### Characteristics of the respondents

Table 1 presents the sociodemographic characteristics of participants. There were 355 respondents involved in the study. Thirty-three percent of the mothers were aged 25 to 29 years, with a mean age of 28.6 ± 6.0 years, and were mostly married (68.2%). Approximately 38.0% of the mothers had completed Junior High School (JHS), and most were engaged in informal work (71.5%). Most of the respondents were Christians (85.9%). The mothers mostly belonged to the Akan (38.1%), and Ga (20.8%) ethnic groups of Ghana. A high proportion of the mothers had female babies (87.6%) and more than half of the babies were aged between 0 and 5 months (58.9%), with a mean age of 5.2 ± 4.1 months. Approximately 96% of all the mothers delivered at the health facility and the proportion of normal/vaginal deliveries was high (78.6%) (Table 1).

**Table 1:** Sociodemographic characteristics of study mothers (*n* = 355)

Characteristics	Frequency	Percent
Age of mothers		
15–19	17	4.8
20–24	74	20.9
25–29	110	33.0
30–34	97	27.2
35–39	41	11.6
40–44	16	4.5
Marital status		
Single	108	30.4
Married	242	68.2
Divorced	5	1.4
Educational level		
No education	38	10.7
Primary	49	13.8
Middle/JHS	133	37.5
SHS/A-level	81	22.8
Tertiary	54	15.2
Ethnicity		
Ga	74	20.8
Akan	135	38.1
Dangme	60	16.9
Ewes	49	13.8
Others^a^	37	10.4
Religion		
Christian	305	85.9
Muslim	50	14.1
Occupation^b^		
Formal employee	101	28.5
Informal employee	254	71.5
Sex of baby		
Male	44	12.4
Female	311	87.6
Age of last baby (in months)		
0–5	209	58.9
6–10	117	32.9
11–15	22	6.2
16–20	5	1.4
21 and above	2	0.6
Place of delivery		
Health facility	341	96.1
At home	14	3.9
Type of delivery		
Normal/Vaginal delivery	279	78.6
Caesarean section	76	21.4

^a^ ethnic groups in northern Ghana

^b^ formal employee: on a monthly salary job & informal employee: not on a monthly salary job

*JHS* Junior High School, *SHS* Senior High School

### Maternal knowledge on breastfeeding

Table 2 provides information on maternal knowledge on breastfeeding practices. There was a universal awareness (100%) of exclusive breastfeeding (EBF), and about 93% correctly indicated that EBF entails feeding an infant with only breast milk. Furthermore, most of the mothers (86.5%) were aware that EBF should span over a period of 6 months, and correctly indicated that initiation of breastfeeding should be within the first hour after delivery (71%). Most of the mothers indicated that water (68.7%) and complementary feed (liquid/solid foods) (81.2%) should be given to babies when they are 6 months old. A high proportion of the mothers (90.7%) identified the health facility as their source of information on breastfeeding (Table 2).

**Table 2:** Maternal knowledge and practices on breastfeeding (*n* = 355)

Statements	Distribution	Percent
Ever heard of exclusive breastfeeding	355	100.0
EBF entails feeding an infant with only breast milk	330	93.0
When a mother should start breastfeeding after delivery		
Within the first hour	252	71.0
Within 24 h	74	20.9
Don’t know	29	8.1
Period for exclusive breastfeeding		
1 month	5	1.4
2 months	6	1.7
3 months	9	2.5
4 months	22	6.2
5 months	6	1.7
6 months	307	86.5
Age at which baby should be given water		
1 month	7	2.0
2 months	38	10.7
3 months	48	13.5
4 months	10	2.8
5 months	8	2.3
6 months	244	68.7
Age at which baby should be given liquid/solid foods		
1 month	4	1.1
2 months	4	1.1
3 months	21	5.9
4 months	34	9.6
5 months	4	1.1
6 months and above	288	81.1
Source of information on breastfeeding		
Health facility	322	90.7
Own mothers	25	7.0
Other relatives	8	2.3

### Breastfeeding practices among mothers

Table 3 presents breastfeeding practices among mothers. Seventy-four percent (95% CI 69.5%, 78.7%) of the mothers were currently breastfeeding their children. More than half of all mothers (63.4%) (95% CI 55.7%, 76.5%) started breastfeeding within the first hour after delivery, and about 81% of all mothers (95% CI 74.5%, 85.7%) offered colostrum to babies after delivery. Exclusive breastfeeding rate under 6 months was 66.0% (95% CI 59.6%, 72.5%) and the continuous breastfeeding rate at 1 year was 77.3% (95% CI 58.3%, 96.3%). Only 33.7% (95% CI 23.9%, 43.5%) of the infants aged 6–8 months were introduced to complementary feeding and among infants less than 24 months receiving any solid food or liquid, less than half (30.1%) were from a bottle (95% CI 25.1%, 35.1%) (Table 3).

**Table 3:** Breastfeeding practices among mothers

Feeding practices	Frequency	Percent	95% CI
Currently breastfeeding (*n* = 355)	263	74.1	69.5, 78.7
Early initiation of breastfeeding (*n* = 355)	225	63.4	55.7, 76.5
Offer colostrum to baby after delivery (*n* = 355)	289	81.4	74.5, 85.7
Exclusive breastfeeding under 6 months (*n* = 209)	138	66.0	59.6, 72.5
Time complementary feeding (*n* = 92)	31	33.7	23.9, 43.5
Continuous breastfeeding at 1 year (*n* = 22)	17	77.3	58.3, 96.3
Bottle feeding (*n* = 326)	98	30.1	25.1, 35.1

### Univariate and multiple regression analysis

Table 4 presents the univariate and multiple logistic regressions of sociodemographic factors associated with exclusive breastfeeding. In the multiple logistic regression analysis using variables that were significant at *p* < 0.05 at the univariate analysis, mothers’ age, education and ethnicity were independently associated with exclusive breastfeeding. Mothers aged 20–24 (AOR 9.80; 95% CI 2.11, 45.46), 25–29 (AOR 9.49; 95% CI 2.07, 43.47), and 30–34 (AOR 6.02; 95% CI 1.41, 25.65) were more likely to practice EBF compared to those aged 15–19 years. Mothers with a tertiary education were less likely to engage in exclusive breastfeeding than those with no education (AOR 0.18; 95% CI 0.36, 0.85). Mothers from ethnic groups in northern Ghana were less likely to exclusively breastfeed their infants compared to those from the Ga ethnic group (AOR 0.29; 95% CI 0.09, 0.96) (Table 4).

**Table 4:** Sociodemographic factors associated with exclusive breastfeeding among mothers (*n* = 209)

Factors	Unadjusted OR	(95% CI)	Adjusted OR	(95% CI)
Age of respondent				
15–19	1		1	
20–24	10.73**	2.92, 39.45	9.80*	2.11, 45.46
25–29	4.21*	1.30, 13.58	9.49*	2.07, 43.47
30–34	4.84*	1.43, 16.40	6.02*	1.41, 25.65
35–39	1.54	0.37, 6.45	1.18	0.25, 5.62
40–44	5.50	1.15, 26.41	2.65	0.44, 15.83
Marital status				
Single	1			
Married	1.28	0.70, 2.33	–	–
Educational status				
No education	1		1	
Primary	1.35	0.42, 4.33	1.35	0.36, 5.15
Middle/JSS	0.77	0.30, 1.97	1.61	0.50, 5.21
SSS/A-Level	3.88*	1.02, 14.76	3.30	0.72, 15.19
Tertiary	0.19*	0.05, 0.67	0.18*	0.36, 0.85
Ethnicity				
Ga	1		1	
Akan	0.38*	0.17, 0.84	0.66	0.23, 1.86
Dangbe	1.77	0.62, 5.08	2.56	0.69, 9.51
Ewes	3.74	0.96, 14.62	2.66	0.58, 12.30
Others	0.33*	0.12, 0.93	0.29*	0.09, 0.96
Religion				
Christian	1			
Muslim	1.52	0.57, 4.05	–	–
Occupation				
Formal employee	1			
Informal employee	0.79	0.41, 1.56	–	–
Place of delivery				
Health facility	1			
At home	1.57	0.31, 7.98	–	–
Type of delivery				
Vaginal delivery	1			
Caesarean section	0.68	0.34, 1.35	–	–
Age of baby (in months)				
0–1	1			
2–3	1.44	0.72, 2.88	–	–
4–5	0.82	0.37, 1.83	–	–
Sex of baby				
Male	1			
Female	1.03	0.44, 2.45	–	–

*OR* Odds Ratio, *CI* Confidence Interval

*p < 0.05, **p < 0.001

## Discussion

Recognizing the important health benefits of breastfeeding based on the adequate evidence available, this study was aimed at assessing maternal knowledge about exclusive breastfeeding, the breastfeeding practices among mothers and the sociodemographic factors that influence exclusive breastfeeding.

There was a universal awareness about EBF and majority of mothers were knowledgeable about exclusive breastfeeding. Similar high awareness and knowledge on EBF has also been reported in Nigeria where 95.3% of mothers had heard about EBF and 82.0% of them correctly defined exclusive breastfeeding [29]. In contrast, a similar study in Nigeria has reported low knowledge about EBF where only 30% (*n* = 179) of the mothers were adequately informed [30]. The high level seen in our study could be due to the effectiveness of the health education and awareness programmes run by the Ministry of Health and Ghana Health Service, and their partners on exclusive breastfeeding. In relation to accessing help and information on breastfeeding, this current study found that mothers tend to go to the health facilities and rely on midwives/nurses for information on breastfeeding. This result is in agreement with the findings of another study in Ghana where all the mothers received information on breastfeeding from health professionals on their visit to health facilities [31]. Oche et al. have on the other hand reported that younger (newer) mothers also mostly rely on their older experienced mothers for information on exclusive breastfeeding [30]. Our current finding stresses the trust and confidence mothers have in the health professionals and the importance of health education to be carried out at the antenatal and postnatal care centres.

Although there was a universal awareness and high level of knowledge about exclusive breastfeeding, only 66.0% of infants less than 6 months were exclusively breastfed. This finding is similar to EBF rate (64.0%) documented by another study in Ghana among infants aged 0–5 months [22] and in Ethiopia where a EBF rate of 60.8% has been reported [32]. However, lower EBF rates of 16.4% and 31.0% have also been reported in similar studies in Nigeria [14, 30]. The discordance between high knowledge and the suboptimal practice of EBF observed in the current study has also been reported by Onah et al. [29]. As stipulated by Onah et al., awareness and knowledge do not necessary translate into practice, probably due to mothers’ lack of appreciation of the vital benefits of EBF, and to address this, strategies should for now focus on practically assisting mothers to addressing the identified challenges to practice of EBF rather than just giving out information on exclusive breastfeeding [29].

This study found factors such as mother’s age, educational status and ethnicity to be associated with exclusive breastfeeding among mothers. The odds of exclusive breastfeeding were high when mothers were older than 20 years. A similar finding has also been reported by Asemahagn where mothers aged 30 years and above were more likely to engage in exclusive breastfeeding [33]. This could be explained as argued by Asemahagn that mothers gain experience in child management as they increase in age, and in the quest of younger mothers to maintain their breast size and beauty, EBF will not practiced for a longer time and introduce early supplementary feeding [33].

In relation to education as a factor of EBF, mothers in our current study who had a tertiary education were less likely to exclusively breastfeed their infants. This finding is in contrast to what has been documented in other similar studies where mothers who had informal education and/or with lower education were all less likely to exclusively breastfeed their infants [29, 33]. Our results could be explained by the fact that in Ghana mothers with higher education tend to engage in formal employment and as observed by Safari et al., mothers who are engaged in formal employment are less likely to exclusive breastfeed [34].

The ethnic affiliation of the mother was found to influence their practice of exclusive breastfeeding. A similar finding has been reported in a similar study by Jacdonmi et al. in Nigeria [35]. The influence of ethnicity on exclusive breastfeeding could be explained by the influence of cultural beliefs and practices of the various ethnic groups in Ghana on breastfeeding practices. For instance, Tawiah-Agyemang and colleagues have postulated that mothers mostly from the Northern ethnic groups in Ghana held the belief that the breast is filled with breast milk on the third day after birth which delays the initiation of breastfeeding [36].

This study also found an early initiation of breastfeeding rate of 63.4% similar to other published study where 57.0% of mothers started breastfeeding within the first hour after birth [6]. This is higher than what has been documented by other studies in Ghana [37], in Ethiopia [38] and in India [39] where timely initiation breastfeeding rates were 39.9%, 52.4% and 23.5% respectively. However, the study found that about 81% of all mothers fed their babies with the first breast milk (colostrum). This is supported by other studies reporting 91.0% in Nepal [6] and 83.3% in Ethiopia [18]. The high rates recorded in our current study could be as a result of the fact that most of the mothers delivered at the health facility, where mothers are encouraged by health personnel to breastfeed just after delivery.

There is widespread and long duration of breastfeeding noted in Ghana [10]. Our study found current breastfeeding and continuous breastfeeding at 1 year of age rates of 74.1% and 76.2% respectively. However, higher rates for current breastfeeding (89.8%) and continuous breastfeeding (89.4%) have been reported elsewhere in Asia [39]. The observed difference could be due to the study design and the sample size differences.

Our study further found that, only 33.7% of infants aged between 6 and 8 months were receiving timely complementary feeding. In contrast to this finding, high timely complementary feeding rates have been documented in Ghana (72.6%) by Issaka et al. [40] and in Ethiopia (60.5%) [41]. As observed by a study in Bangladesh, where mothers with low education were less likely to timely introduce complementary feeding among infant aged 6–8 months [42], the low educational background of most of the mothers in our study could explain the lower rate of timely complementary feeding. This suggests the need for an intense education on the importance of complementary feeding among mothers.

Even though bottle feeding is reportedly not widespread in Ghana [10], bottle feeding among infants less than 24 months was 30.1%. This was higher than what has been reported elsewhere (14.8%) [39]. As found by Petal et al. where high bottle feeding was found among urban and wealthier mothers [39], the high rate of bottle feeding reported in the current study could be explained by the fact that the study was done in a peri-urban community with characteristics similar or close to urban counterparts. These current findings suggest the need for an intense campaign on the importance of timely, adequate and safe complementary feeding and interventions necessary to bring down the high rates of bottle feeding among mothers.

The results of this study are limited. The findings of the study cannot be generalized for Greater Accra Region and other similar setting as the study respondents were recruited from a single health centre in the Tema East Sub-Metropolitan area of the Greater Accra Region but could be considered indicative of the context considered.

## Conclusions

Mothers were adequately informed about exclusive breastfeeding. The practice of exclusive breastfeeding among infants less than six months old in the previous 24 h and complementary feeding among infants aged 6–8 months were suboptimal. Exclusive breastfeeding among mothers was influenced by mother’s age, ethnicity and educational status. Interventions emphasizing practical education should therefore be targeted at addressing factors that influence exclusive breastfeeding.

## Abbreviations

CWC: Child welfare clinic
EBF: Exclusive breastfeeding
ERC: Ethical review committee
GHS: Ghana Health Service
JHS: Junior high school
OR: Odd ratio
SHS: Senior high school
